# Isolation of a novel monkey adenovirus reveals a new phylogenetic clade in the evolutionary history of simian adenoviruses

**DOI:** 10.1186/1743-422X-8-125

**Published:** 2011-03-17

**Authors:** Carlos Maluquer de Motes, Ayalkibet Hundesa, Francisca C Almeida, Silvia Bofill-Mas, Rosina Girones

**Affiliations:** 1 Section of Virology, Faculty of Medicine, Imperial College London, Norfolk Place W2 1PG, London, UK; 2 Department of Microbiology, University of Barcelona, Diagonal 645, 08028 Barcelona, Spain; 3 Department of Genetics, University of Barcelona, Diagonal 645, 08028 Barcelona, Spain

## Abstract

Adenoviruses of primates include human (HAdV) and simian (SAdV) isolates classified into 8 species (Human Adenovirus A to G, and Simian Adenovirus A). In this study, a novel adenovirus was isolated from a colony of cynomolgus macaques (*Macaca fascicularis*) and subcultured in VERO cells. Its complete genome was purified and a region encompassing the hexon gene, the protease gene, the DNA binding protein (DBP) and the 100 kDa protein was amplified by PCR and sequenced by primer walking. Sequence analysis of these four genes showed that the new isolate had 80% identity to other primate adenoviruses and lacked recombination events. The study of the evolutionary relationships of this new monkey AdV based on the combined sequences of the four genes supported a close relationship to SAdV-3 and SAdV-6, lineages isolated from *Rhesus *monkeys. The clade formed by these three types is separated from the remaining clades and establishes a novel branch that is related to species HAdV-A, F and G. However, the genetic distance corresponding to the newly isolated monkey AdV considerably differs from these as to belong to a new, not yet established species. Results presented here widen our knowledge on SAdV and represents an important contribution to the understanding of the evolutionary history of primate adenoviruses.

## Findings

Adenoviruses are non-enveloped, icosahedral, double-stranded DNA viruses known to cause gastroenteritis, keratoconjunctivitis, and acute respiratory disorders [1]. Adenoviruses infecting primates belong to the genus *Mastadenovirus*. To date, at least 52 distinct human adenovirus (HAdV) types have been described. Phylogenetically, HAdVs cluster into 6 species (named Human Adenovirus A to F, HAdV-A to F), although recently a new species (HAdV-G) has been described to include the types HAdV-52 and SAdV-1 [2]. Moreover, at least 25 simian adenovirus (SAdV) types have been recognized: 20 types (SAdV-1 to 20) were isolated from Old World monkeys and are more related to species HAdV-A and F [3,4], whereas 5 types (SAdV-21 to 25) were isolated from chimpanzees and are closely related to HAdV-4 (HAdV-E) and to HAdV-B [5-7]. Recently, new isolates from chimpanzee, bonobo, orangutan, gorillas, and macaques have been described and characterized, largely expanding the SAdV taxonomy [8,9].

HAdVs have traditionally been classified according to their immunochemical and biological properties. Nowadays, however, microbiologists favour to classify HAdVs based on their evolutionary relationships as inferred in phylogenetic analyses of DNA sequences of viral protein genes. This approach allows for a classification based on the evolutionary history of the viruses, besides avoiding misleading classification due to cross-reaction in neutralization and hemagglutination tests. In addition to the γ determinant of the fiber protein, the main type-specific epitope in adenovirus is the ε determinant present on the hexon capsid protein. DNA amplification and sequencing of these informative regions in the adenoviral genome have been used to provide molecular data for typing strains and identification of new prototypic isolates [10,11]. Moreover, the hexon and the protease genes have been characterized in most adenovirus types providing a large amount of data for taxonomic classification of new isolates.

**Figure 1:**
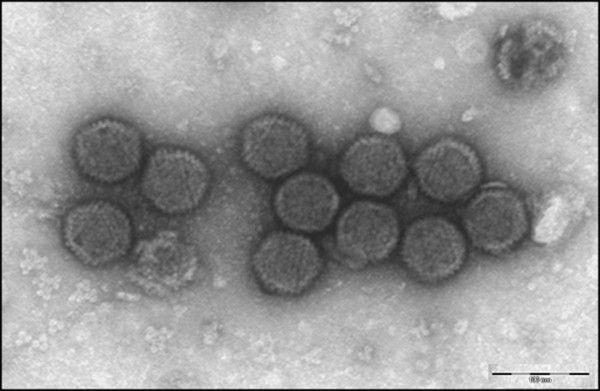
**Transmission electron micrograph of the newly isolated viral particles present in the supernatant of Vero cells infected with a viral concentrate obtained from cynomolgus monkeys**. Bar, 100 nm.

In this study we describe the isolation and identification of an adenoviral type strain from cages inhabited by a colony of cynomolgus monkeys (*Macaca fascicularis*). Viral concentrates were obtained from a mix of excreta and waste collected from bedding areas of a colony of *M. fascicularis *showing no signs of gastroenteritis or pathogenic disorders. Viruses were eluted and concentrated from the samples as described previously [12]. African green monkey kidney epithelial cells (Vero) were infected blind with the viral concentrates and cytopathic effect was rapidly observed. Supernatant from infected Vero cells was prepared for analysis by transmission electron microscopy using copper grids coated with formvar film and carbon that were negatively stained with 2% phosphotungstic acid, pH 7.0. Analysis of the grids using a JEOL JEM-1010 identified adenoviral particles characterized by typical 70 nm icosahedral-shaped capsids (Figure 1). The virus (hereafter referred to as CynAdV) was purified by infection of end-point limiting dilutions in Vero cells for 3 passages. A purified stock was generated and the virus titer was determined as 10^7.5 ^TCID_50 _ml^-1 ^by calculation using the Reed-Muench cumulative method.

The entire viral DNA genome was purified as described by Kovács et al. [13]. A random library of DNA fragments was prepared and some clones were sequenced. To address the identification of the new isolate, a genome fragment encompassing traditionally studied viral genes like the hexon and the protease gene was targeted for amplification and sequencing. Two fragments of approximately 3,500 bp covering the whole coding sequence for the hexon, protease, DBP genes, and part of the 100 kDa gene were then amplified with FideliTaq (Amersham Biosciences) following the manufacturer's instructions. The fragments were purified with the QIAquick PCR purification kit (QIAgen) and sequenced with the ABI PRISM Dye Terminator Cycle Sequencing Ready Reaction kit (Applied Biosystems) using primer walking. The sequence was deposited in the GenBank with accession number EU293065.

The CynAdV genome contained a 2,775 bp-long hexon gene, a 615 bp-long protease gene, and a 1,320 bp-long DBP gene. The overall base composition of the 7,481 bp sequence was 21.43% A, 21.61% T, 26.55% G, and 30.41% C. The GC content was 56.96%, similar to those reported for the complete genome of HAdV-52 (55.1%) and SAdV-1 (55%). Interestingly, no intergenic region was observed between the hexon and protease genes, suggesting that the CynAdV genome size might be smaller than other SAdVs. When the predicted amino acid sequences were used as queries in BLASTp searches, CynAdV showed an overall amino acid identity of 75-88% for the hexon protein, a structural protein with long conserved domains (Table 1). Amino acid identity was reduced in the non-structural proteins, protease and DBP. The CynAdV protease consisted of a protein of 205 residues and showed 75-80% amino acid identity to most human and simian adenovirus protease sequences, whereas the DBP consisted of 493 residues and showed 46-59% amino acid identity to most adenoviral DBP sequences (Table 1).

**Table 1:** Amino acid identity among the predicted protein sequences for hexon, hexon region L1, hexon region L2, protease and DNA binding protein (DBP) of CynAdV and those of different HAdV and SAdV types

			Amino acid identity to CynAdV (%)				
**AdV species/serotype**	**Species of origin**	**Accession Number**	**Hexon**			**Protease**	**DBP**
			**Hexon**	**L1**	**L2**		

**HAdV-A**							
HAdV-12	*Homo sapiens*	NC_001460	83	54	51	75	52
**HAdV-B**							
HAdV-7	*Homo sapiens*	AC_000018	77	41	41	78	52
HAdV-11	*Homo sapiens*	NC_004001	78	48	32	77	49
HAdV-35	*Homo sapiens*	AC_000019	77	46	37	77	49
SAdV-21	*Pan paniscus*	AC_000010	77	46	42	81	52
**HAdV-C**							
HAdV-1	*Homo sapiens*	AC_000017	76	47	54	75	46
HAdV-2	*Homo sapiens*	NC_001407	77	51	52	75	49
HAdV-5	*Homo sapiens*	AC_000008	76	47	39	75	46
**HAdV-D**							
HAdV-9	*Homo sapiens*	NC_010956	79	42	37	78	50
HAdV-26	*Homo sapiens*	EF153474	77	36	34	78	50
HAdV-53	*Homo sapiens*	NC_002067	78	38	34	78	49
**HAdV-E**							
HAdV-4	*Homo sapiens*	NC_003266	77	36	43	78	54
SAdV-22	*Pan paniscus*	AY530876	79	40	54	80	53
SAdV-23	*Pan paniscus*	AY530877	78	41	35	80	53
SAdV-24	*Pan paniscus*	AY530878	79	42	57	80	53
SAdV-25	*Pan paniscus*	AC_000011	79	41	60	80	52
**HAdV-F**							
HAdV-40	*Homo sapiens*	NC_001454	82	51	37	76	53
**HAdV-G**							
HAdV-52	*Homo sapiens*	DQ923122	88	65	62	77	55
SAdV-1	*Macaca fascicularis*	NC_006879	85	58	53	78	55
**SAdV-3**	*Macaca mulatta*	NC_006144	78	41	43	79	59
**SAdV-7**	*Macaca mulatta*	DQ792570	87	66	44	77	54

**Figure 2:**
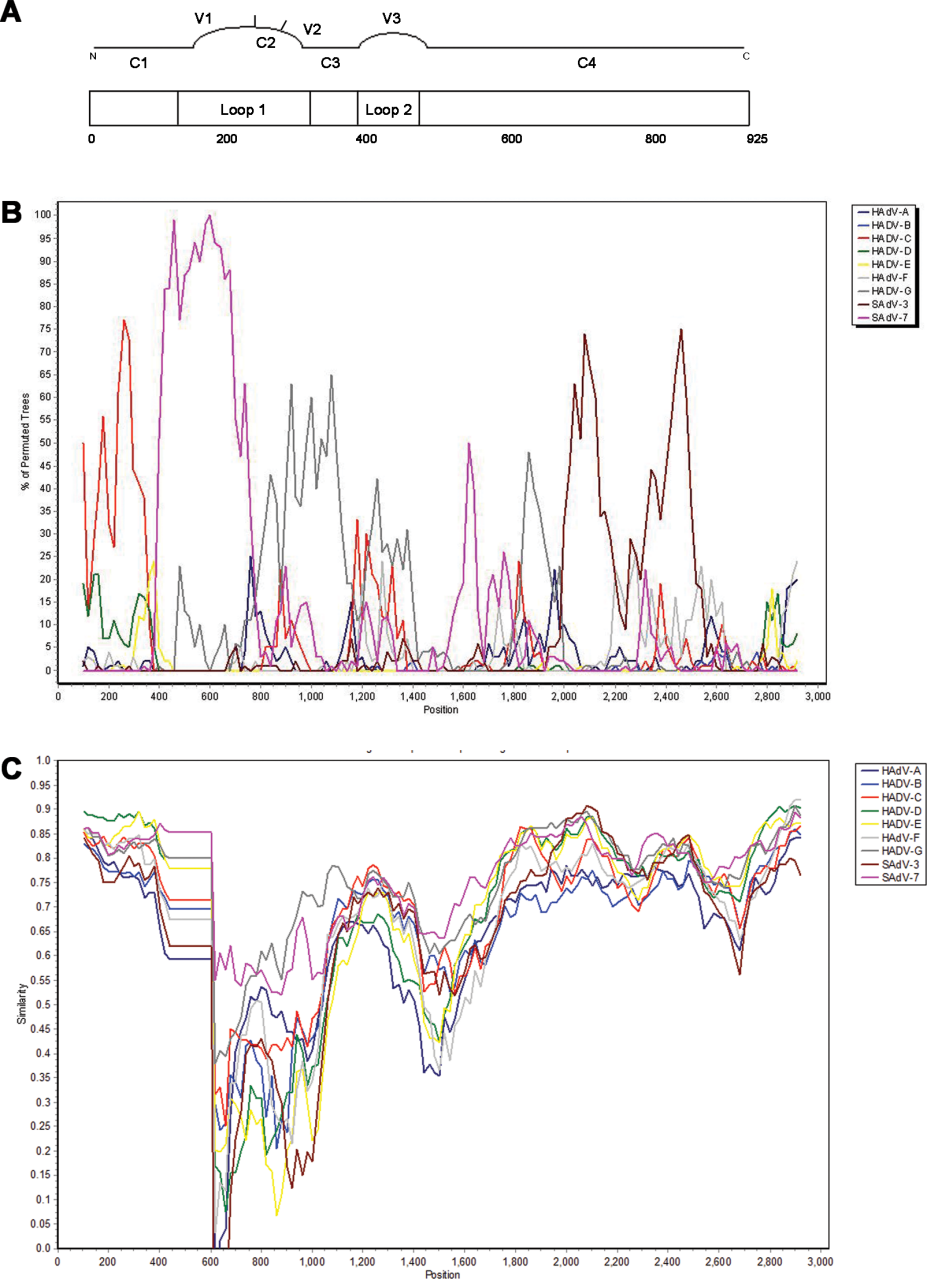
**Recombination analysis in the hexon gene of the newly isolated monkey AdV:** A) schematic representation of the adenoviral hexon gene, B) bootscan analysis and C) Simplot of CynAdV against the hexon genes of described human and simian adenoviruses. The following settings were used: 200-bp window size, 20-bp step size, 100 replicates, gap stripping, Kimura-2 parameter distance model, Neighbour Joining tree model, and parental threshold 85.

Recombination among adenovirus serotypes generates new strains (intermediate types) that are difficult to classify. Due to selective pressure of neutralizing antibodies and viral escape, recombination events involving the hexon are common, as has recently been described [14,15]. The hexon protein in HAdVs can be divided into 4 conserved (C1-4) and 3 variable regions (V1-3) [16]. It contains the type-specific neutralization epitope (ε determinant) which consists of 6 hypervariable regions located in L1 and an extra one located in L2 [17]. High identity of these variable regions with other HAdVs lineages could indicate recombination in this particular gene. To assess whether recombination occurred in the newly isolated monkey AdV hexon gene, we performed bootscan analysis of the prototypic hexon sequences available using SimPlot software [18]. Hexon gene sequences were aligned using ClustalW and the CynAdV sequence was analyzed against those of HAdV and SAdV types grouped according to species. Grouping reduces effects of variation in individual sequences. However, considering the similarity between CynAdV and SAdV-7 and members of the species HAdV-G, these sequences were left ungrouped. The results of the bootscan analysis failed to identify recombination events (Figure 2).

In order to classify the new isolate, the evolutionary relationships of CynAdV were assessed in a phylogenetic analysis including all publicly available genomes of HAdVs and SAdVs. Sequences were aligned with MAFFT [19] using standard gap parameters and 100 refinement iterations. The alignment was then trimmed to match the coding regions of the genes sequenced for CynAdV: hexon, protease, DBP, and 100 kDa genes. Tree searches were performed on each gene individually and on the combined dataset (coding sequences of all 4 genes concatenated) using maximum likelihood methods as implemented in the program RAxML 7.0.4 [20]. For all matrices, the evolutionary model used was the GTRGAMMA and 20 search replicates were performed. In the analysis of the combined dataset, model parameters were obtained for each gene separately (partition wise). Statistical significance of clades was obtained with 100 bootstrap searches. A second analysis of the combined dataset was done after removing highly variable regions including many gaps that could cause low accuracy in the alignment.

**Figure 3:**
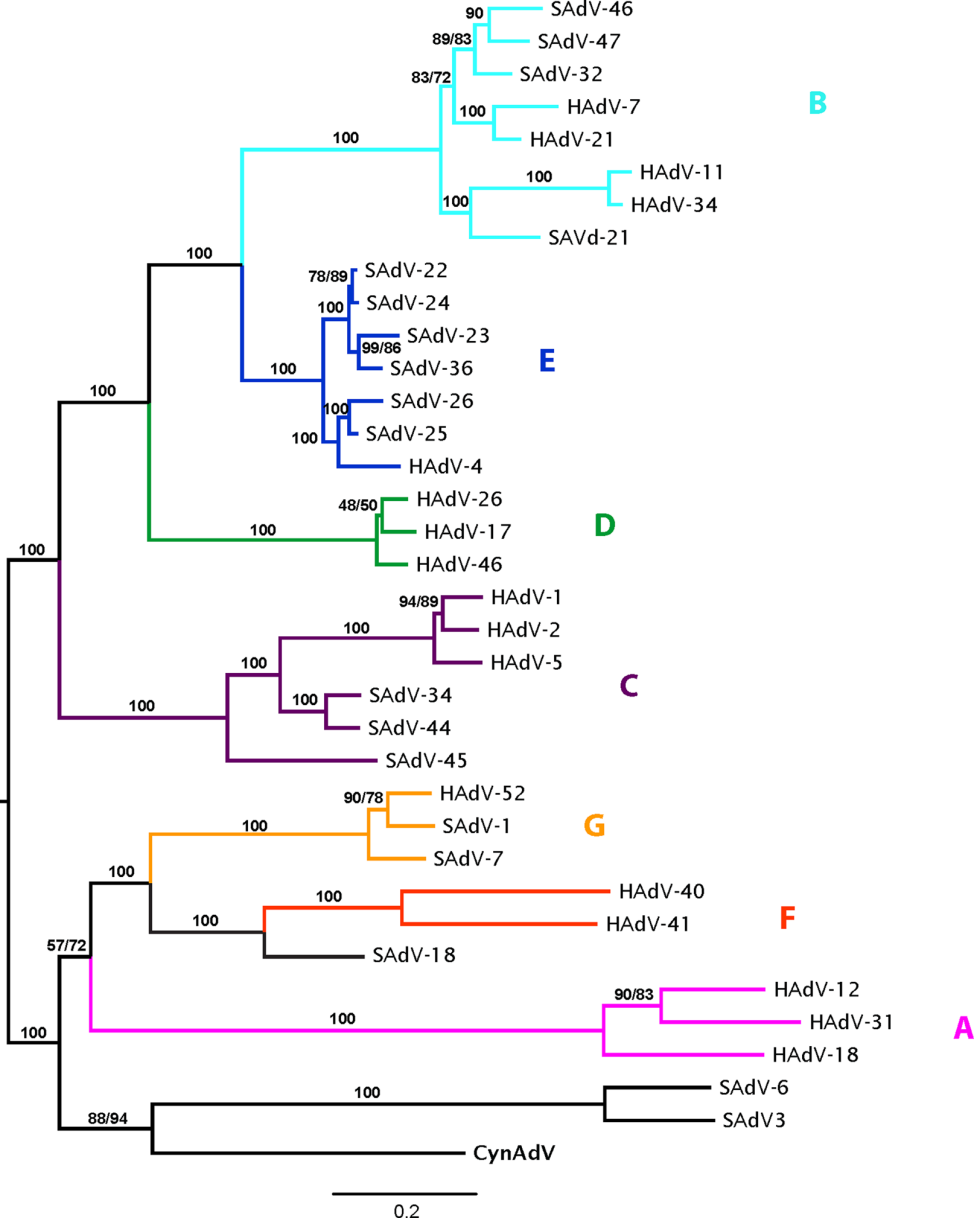
**Phylogenetic analysis of CynAdV**. The tree was constructed from an alignment of a concatenated sequence containing the hexon, protease, DBP and 100 kDa protein, using maximum likelihood methods. Numbers on nodes represent bootstrap values obtained with (left of slash) and without (right of slash) highly variable sites. When only one value is present, both analyses gave the same value.

**Figure 4:**
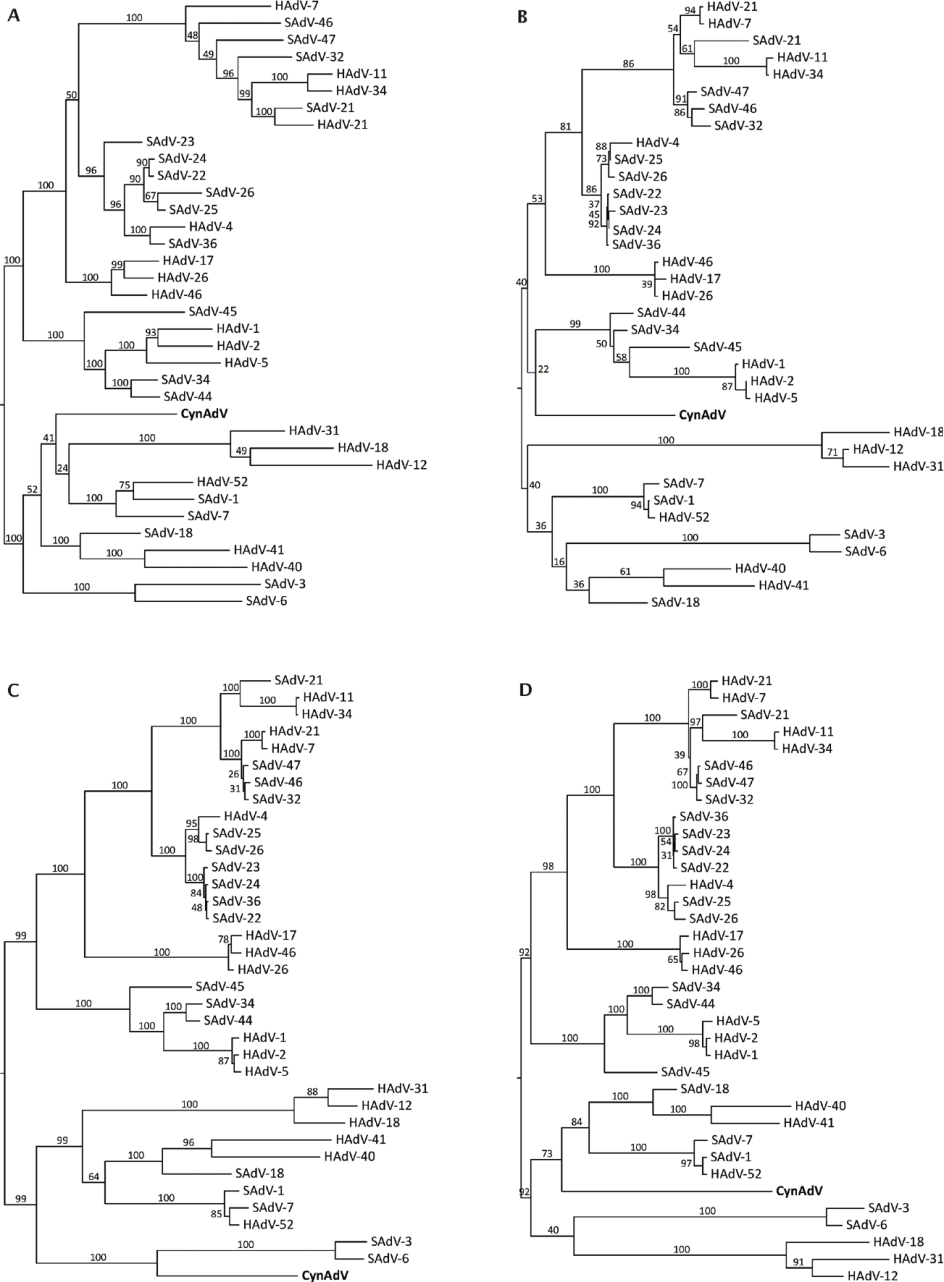
**Individual gene trees obtained in maximum likelihood analysis for:** A) hexon, B) protease, C) DBP, and D) 100 kDa protein. Numbers on notes refer to bootstrap percentages.

**Figure 5:**
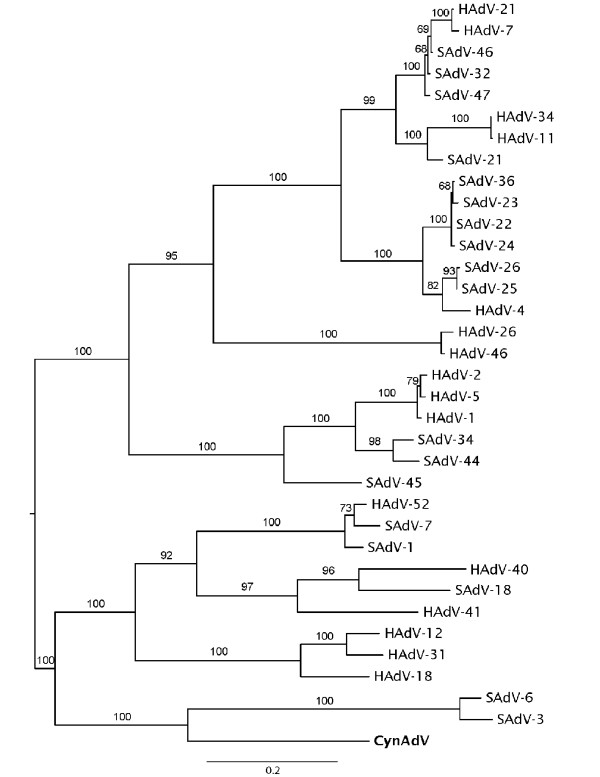
**Maximum likelihood tree obtained with the amino acid sequence of the DBP gene**. Numbers on notes refer to bootstrap percentages.

The resulting trees (combined dataset in Figure 3, individual gene trees in Figure 4) recovered all adenovirus species groups as in Roy et al. [8]. The combined dataset tree had high support for all previously described HAdVs species and for most relationships among groups. In this tree, CynAdV clustered the closest with a clade including species SAdV-3 and SAdV-6 with moderately high bootstrap support (88%). The support for this relationship increased (94%) when highly variable regions were removed (Figure 3). Individual gene trees (Figure 4) disagreed on the relationships among species HAdV-A, F, G, the clade formed by types SAdV-3 and SAdV-6, and CynAdV. The conflicting relationships, however, did not receive high bootstrap support in any of the gene trees. The only exception was the DBP gene tree that agreed and showed high statistical support for the relationships of CynAdV depicted in the combined dataset tree. To check whether high levels of variation in DBP (as illustrated by low sequence identity across species, Table 1) could be biasing the combined analysis [21], we reanalyzed this gene using this time its amino acid sequence. The resulting tree is very similar to the one based on nucleotides, suggesting that this gene is not highly affected by sequence bias and that it is probably a good marker to use in the classification of adenoviruses (Figure 5).

Phylogenetic signal conflict among genes was assessed with the incongruence-length difference (ILD) test [22] as implemented in the software PAUP*. The IDL test showed significant conflict among the phylogenetic signal of individual genes (p = 0.001). One explanation for this conflict is nucleotide substitution saturation, which may blur phylogenetic signal in individual genes [23]. This explanation is supported by the increase in bootstrap percentages when highly variable regions were removed from the combined dataset. An alternative explanation is recombination between an ancestor of the CynAdV virus and other viruses of related species groups A, F, or G. The low bootstrap percentages obtained for CynAdV in individual gene analyses (except for DBP), however, precludes the formulation of a specific hypothesis for the recombination event. As shown before, the test for recombination involving the hexon gene was negative.

In a previous study [8], three different lineages of adenovirus isolated from cynomolgus macaques were identified. Interestingly, these three lineages were also associated to SAdV-3 and SAdV-6 (isolated from *Rhesus *monkeys) in trees based on the DBP gene. Nevertheless, these relationships were not observed for two of the newly described varieties (SAdV-49, and SAdV-50) in trees obtained with other genes. In fact, their results were very similar to ours in that the relationships of the lineages classified as species A, F, and G were largely discordant. Unfortunately, since the sequences of the other cynomolgus macaque's adenoviruses - SAdV-48, SAdV-49, and SAdV-50 - were not publicly available, they could not be included in the phylogenetic analyses presented here.

In the past years, sequencing of the AdV hexon gene has proved to be sufficient for typing new isolates [11,16]. However, phylogenetic data inferred from hexon gene analysis needs to be contrasted against other viral genes since selective pressure on the hexon (and fiber genes) may lead to recombination events and convergent evolution. In this study, analysis of a genomic region encompassing 4 different genes including the hexon gene distinguished the newly isolated monkey AdV as an independent clade close to the types SAdV-6 and SAdV-3. The trees shown here suggest that SAdV-6 may belong to species SAdV-A together with SAdV-3. However, CynAdV seems to adequately differ from these to belong to a separate (not yet established) species. This study represents an important contribution to the understanding of the diversity and evolutionary history of primate adenoviruses.

## Competing interests

The authors declare that they have no competing interests.

## Authors' contributions

CMM and SB collected the sample and isolated the virus. AH cultured the virus and generated purified stocks. CMM and AH purified the viral genome, conceived the amplification and sequencing strategy, and analyzed the sequences obtained. FCA performed the phylogenetic analysis. CMM, FCA and RG analyzed the data and drafted the manuscript. All authors read and approved the final manuscript.
